# Evaluating students’ experiences in self-regulated smart learning environment

**DOI:** 10.1007/s10639-022-11126-0

**Published:** 2022-07-04

**Authors:** Yusufu Gambo, Muhammad Zeeshan Shakir

**Affiliations:** School of Computing, Engineering and Physical Sciences, University of the West of Scotland, Paisley, PA12BE Scotland UK

**Keywords:** Self-regulated learning, Smart learning environment, Online learning, Mobile app, Mixed-method

## Abstract

The increasing development in smart and mobile technologies transforms a learning environment into a smart learning environment that can support diverse learning styles and skills development. An online learner needs to be supported for an engaging and active learning experience. Previously, this progressive research developed and implemented a self-regulated smart learning environment (mobile app) among final-year undergraduate students to support online learning experiences. To understand students' experiences, there is a need to evaluate the mobile app. However, there is a lack of a well-documented study investigating students' experiences in terms of usability, challenges, and factors influencing satisfaction to inform a decision regarding future implementation. This study attempts to fill these gaps by exploring these experiences for sustainable future implementation. The study used cyclical mixed-method evaluations to explore the experiences of 85 final-year undergraduate students. The quantitative data were collected using a survey on the constructs of the research model previously developed to evaluate factors influencing students' satisfaction, and the qualitative used focus group discussions to explore usability experiences and challenges of implementations. The quantitative data were analyzed using SPSS 25 to confirm the structural equation model's relationship. The qualitative data were analyzed using a thematic process to understand students' experiences. The findings from the first mixed-method evaluation show that students were able to follow the learning process, and the application supported their online learning experiences. However, a student expressed the need to improve user functionalities to motivate and engage them in the learning process. The suggestions were incorporated into the mobile app development for the second evaluation. The findings from the second evaluation revealed similar support. However, students suggested a web-based version to support different operating systems and improve interactions. Furthermore, the information system qualities and moderating factors investigated supported students' satisfaction. Future research could explore facilitators' experiences in the mobile app for sustainable development and implementation for engaging online learning experiences and skills development.

## Introduction

The increasing developments in smart and mobile technologies such as artificial intelligence, machine learning, the internet of things (IoT), and wearable computing devices have continued to impact every sphere of life. It is possible to compute anywhere using the superior power of mobile devices connected to the internet (Fakinlede et al., 2015; Kopačková, 2014). As the centre for research, innovations, and development, educational institutions have continued to be more innovative due to these new technology developments. The educational institutions are now called a smart campus, smart education, smart learning environment, smart classroom, and smart learning process as the results of the transformative power of smart and mobile technologies (Spector, 2016; Yot-Dominguez & Marcelo, 2019; Zhu et al., 2016).

Educational institutions saw these opportunities and, coupled with the challenges of the Covid-19 pandemic and infrastructure deficits, are now offering a blended pedagogical framework to meet the need of on-campus and off-campus students and those on remote learning. There is an increasing deployment of skilled-based courses in an online learning environment to meet skills gaps in the developing workforce (Gambo & Shakir, 2019; Hoel & Mason, 2017; Zhu et al., 2016; Zhu & He, 2012). However, an online learner needs to be supported to succeed in the learning process and achieve a learning goal. Self-regulated learning has been identified as one strategy to develop skills needed to support an online learning process (Gambo & Shakir, 2019; Zhu et al., 2016; Zimmerman, 2002). This learning strategy can be integrated into a smart learning environment to provide personalized support for an inclusive learning experience.

Previously, this progressive research developed and implemented a self-regulated smart learning environment (mobile app) among students to support online learning experiences (Authors, 2021b). It's important to investigate students' experiences in the mobile app to inform future implementation decisions. This progressive work previously integrated the constructs of the information system success model (system quality, information quality & service quality) (Delone & McLean, 2003) and social cognitive (environmental, personal & behavioural) (Bandura, 2002) to understand factors influencing students' satisfactions in a self-regulated smart learning environment (Gambo & Shakir, 2019). This is due to the scarcity of a comprehensive research model that provides insights into factors influencing students' satisfaction in a self-regulated-based learning environment. The authors hypothesized that social cognitive constructs could moderate information system qualities to impact students' satisfaction; and called for numerical evidence in a real contextual setting.

In an attempt to address the need to provide numerical evidence of the relationships of the constructs in the evaluation model of the self-regulated smart learning environment and factors influencing students' satisfaction (Gambo & Shakir, 2019), this study explores students' experiences in the mobile app to inform future implementation decisions. Thus, the emerging question is: What are the experiences of students in self-regulated smart learning environments and sub-questions are:

i. What factors influence students' satisfaction in the self-regulated smart learning environment?
ii. Were students able to follow the self-regulated smart learning environment process?
iii. What functionalities of the self-regulated smart learning environment need to be improved to enhance online learning experiences?
iv. What are the challenges in using the self-regulated smart learning environment?

## Background information and related works

### Overview of the self-regulated smart learning environment (mobile app)

The self-regulated smart learning environment (mobile app) is an Artificial intelligence-based smart learning environment supported by the Zimmerman self-regulated learning model as the pedagogical process that aims to develop skills and content knowledge to support online learning. The purpose is to provide motivation, engagement, active learning and support through learning personalization based on the learner's learning behaviour (Authors, 2021a).

**Fig. 1:**
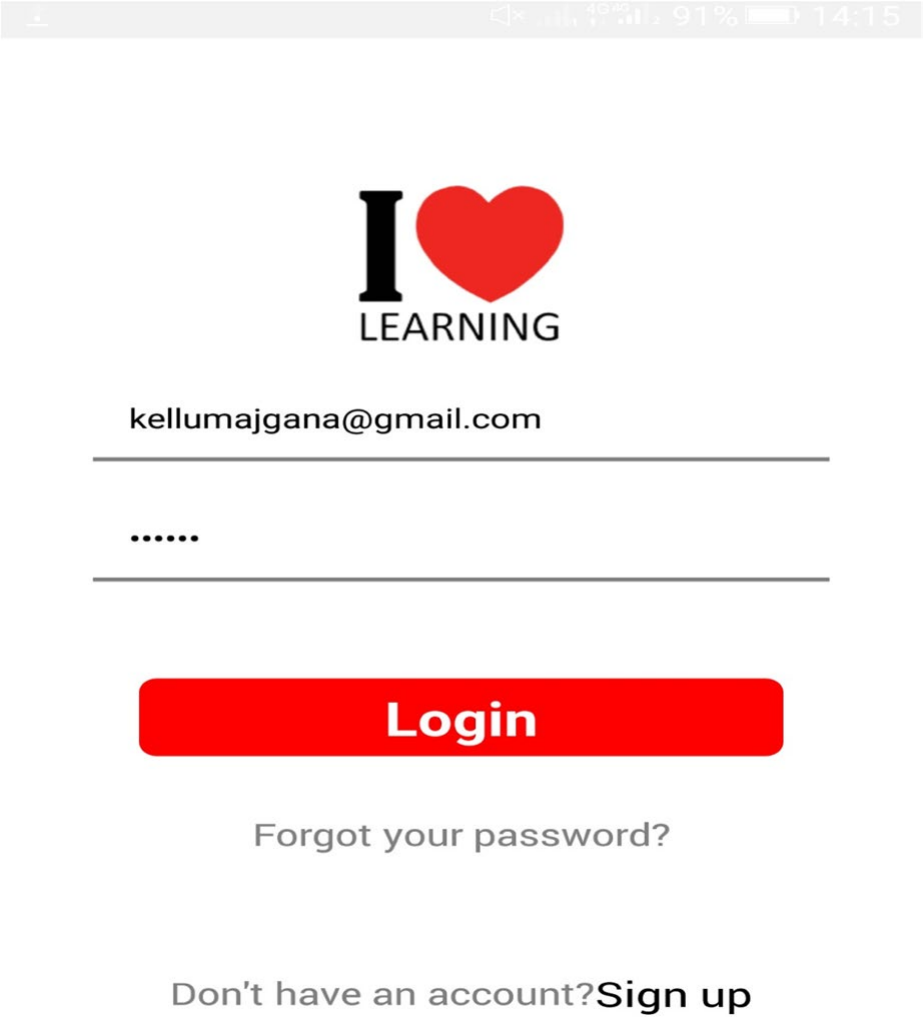
Sign up

**Fig. 2:**
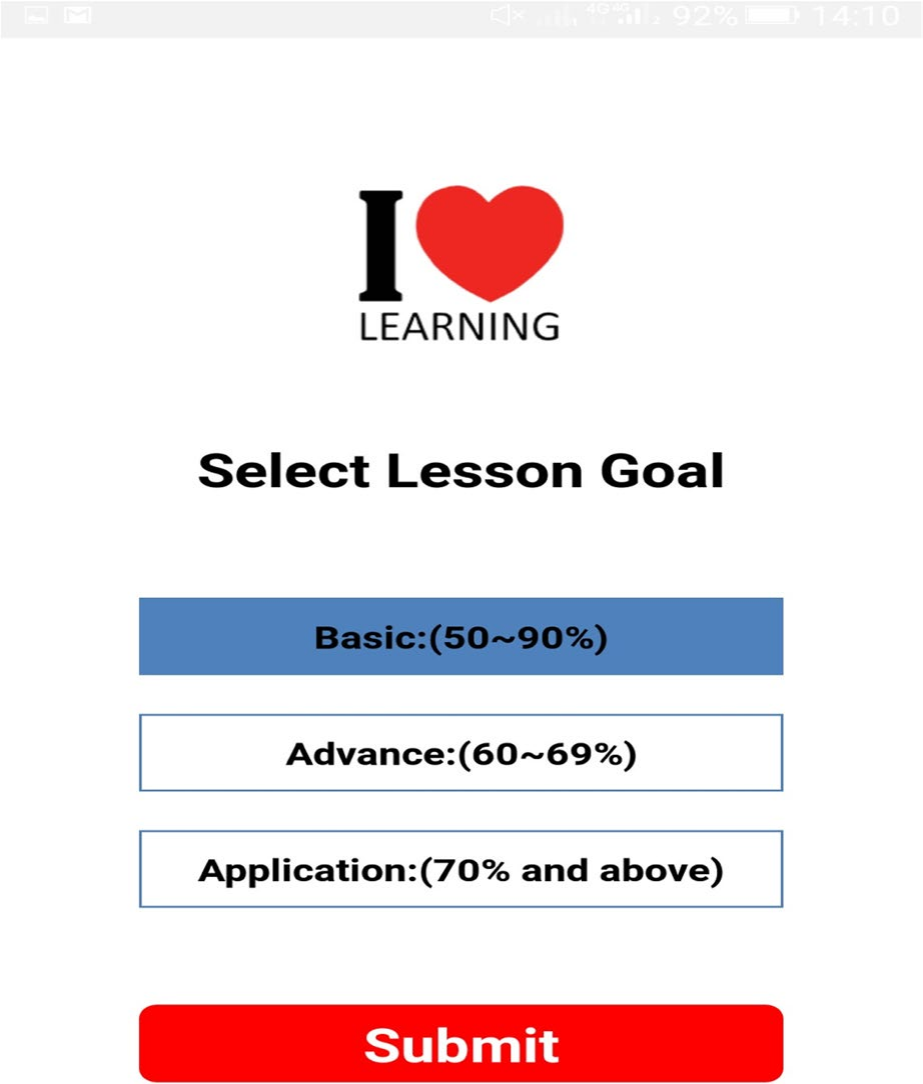
Goal selection

**Fig. 3:**
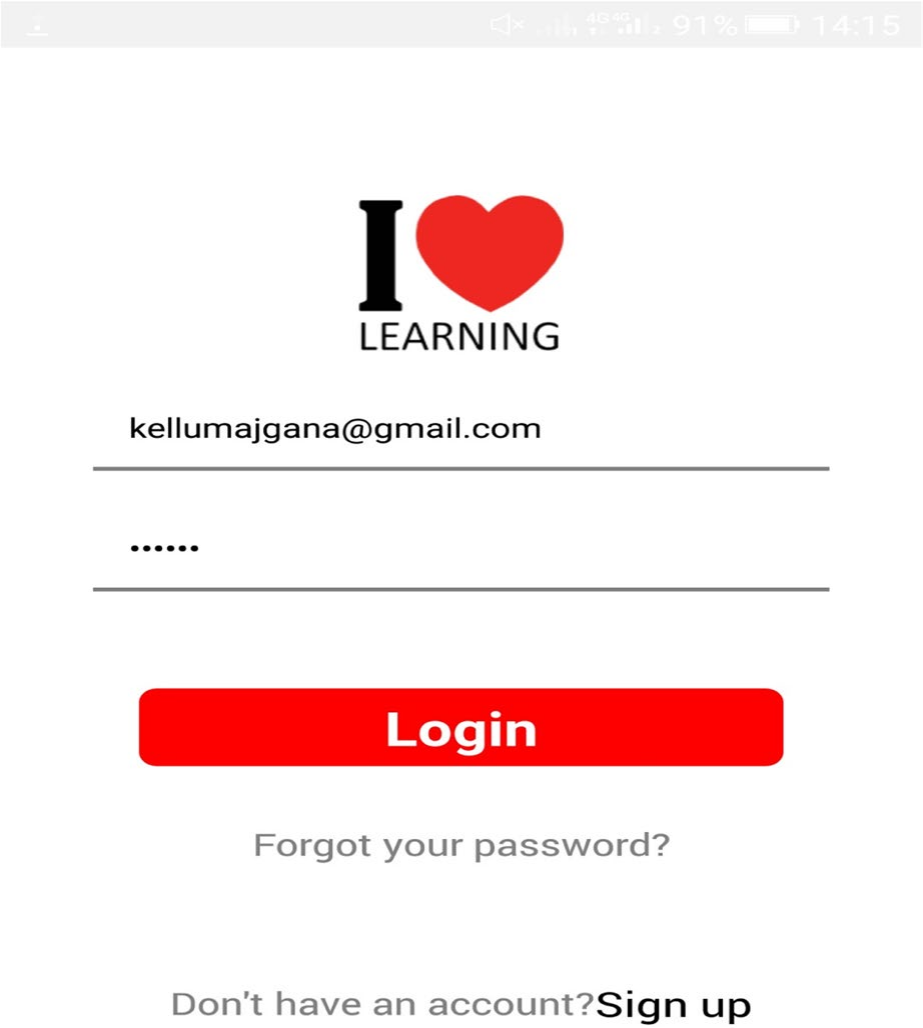
Login

The mobile app integrated self-regulated learning processes (forethought phase: where learners access knowledge and set learning goals). The selection of a learning goal (Basic, advanced & Application) is what the learner aims to achieve after using the learning resources and help-seeking facility to support online learning experiences. The login process and goal selection are shown in Figs. 1, 2, and 3.

**Fig. 4:**
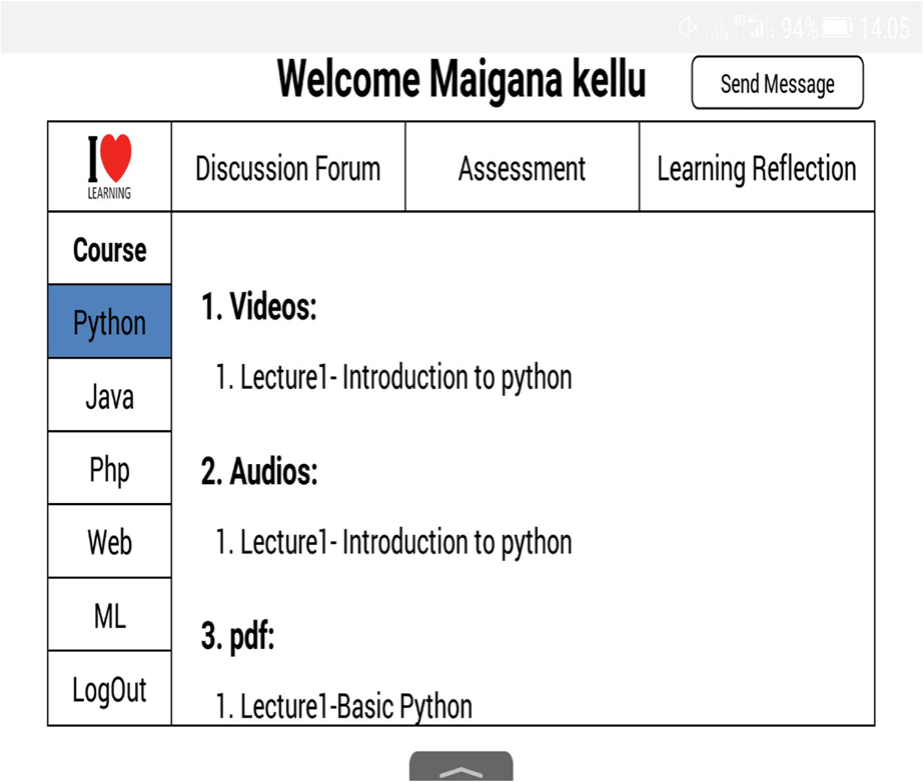
Learning resources

**Fig. 5:**
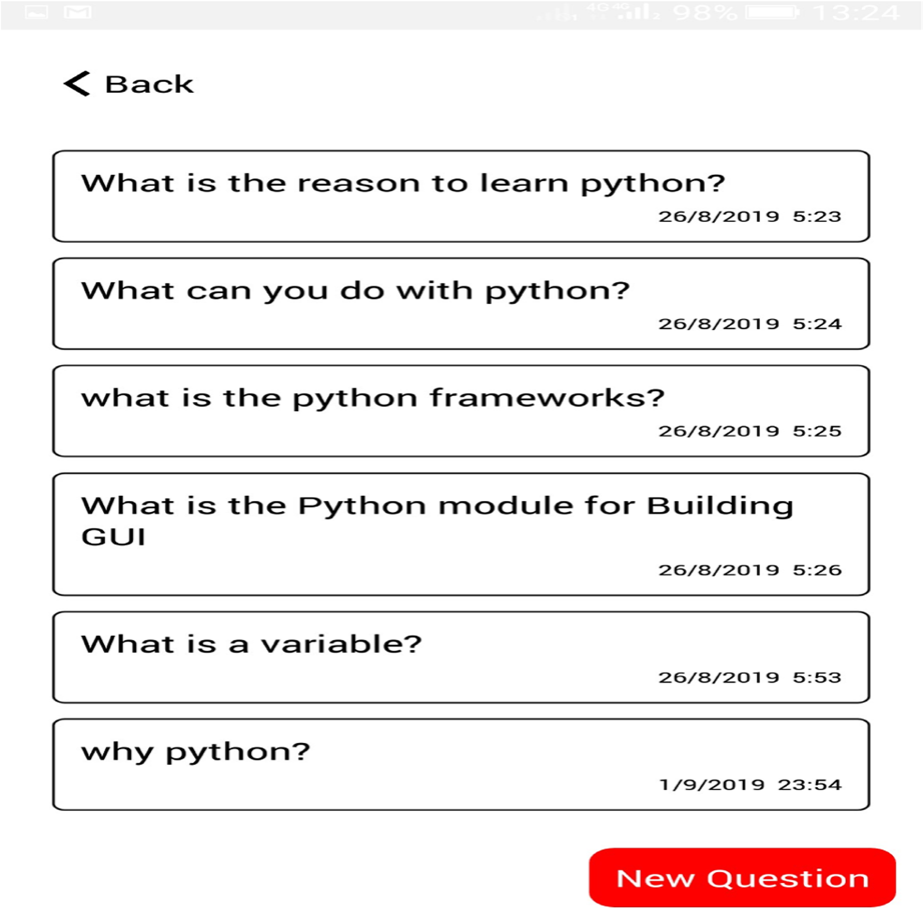
Help-seeking

**Fig. 6:**
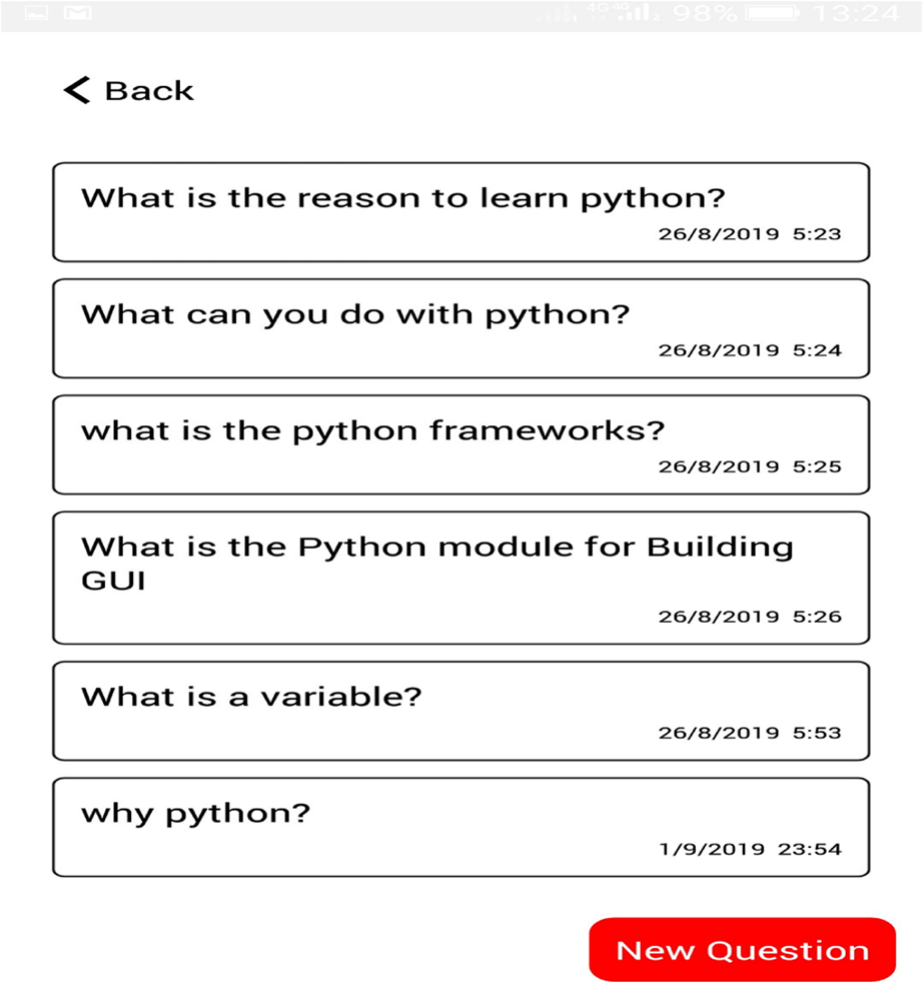
Help-seeking

The performance phase allows students to access learning resources, plan for task strategies, and use discussion forums for help-seeking. The learning contents are in different formats based on the learning preference of a learner, i.e. videos, pdf and audio; and the help-seeking is a chatting window that allows a student to interact with peers or tutors for information about the learning contents or additional resources as shown in Figs. 4, 5, and 6.

**Fig. 7:**
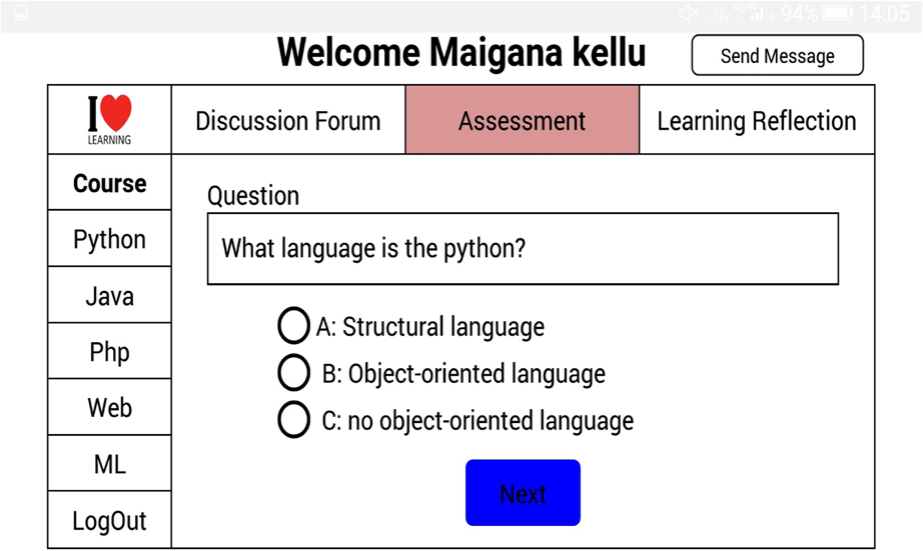
Assessment table

**Fig. 8:**
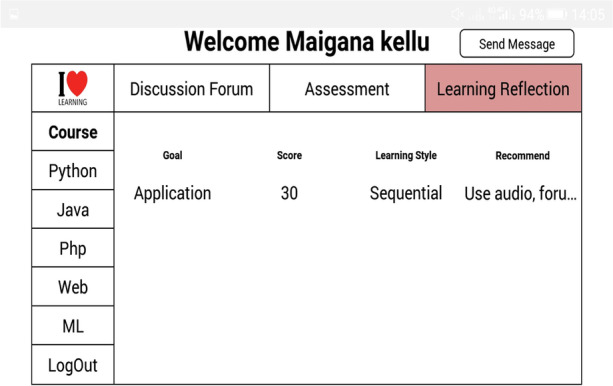
Learning reflection

The self-reflective, where students assess and evaluate their learning performances to support learning skills (goal setting, task strategy, help-seeking, time management, and self-evaluation) and knowledge content to enhance online learning experiences. The assessment tab allows students to take a random quiz based on the selected learning goal. The learning reflection allows students to monitor their learning process, style and personalized learning resources based on their learning behaviours, as shown in Figs. 7 and 8.

This mobile app has been implemented among undergraduate students. This study evaluates their experiences in terms of challenges, interface usability and factors influencing satisfaction to inform pedagogical design principles for future implementations.

### Related works

Studies evaluating technology-enhanced learning can be carried out at the pre-implementation, implementation, and post-implementation stages (Alawani & Singh, 2017; Guerra et al., 2016; Sasai, 2017). Evaluating technology-enhanced learning provides insights to educational stakeholders about why learning technology fails or succeeds and how best it can be implemented for effective pedagogical delivery. However, most evaluations in technology-enhanced learning studies are carried out during the post-implementation stage to determine the impact of technology on user experiences as well as the organization (Alkhasawnh & Alqahtani, 2019; Friedman & Wyatt, 1997; Guerra et al., 2016; Sasai, 2017). Thus, technology-enhanced learning and evaluation of system implementation is an important endeavour, evident through many publications (Guerra et al., 2016; Nussbaumer et al., 2015; Mohammed & Garibaldi, 2010; Sasai, 2017). Friedman and Wyatt (1997) noted that there are two types of evaluation studies: formative and summative. The formative evaluation aims to improve the technology by providing feedback to the developers or implementers. The summative evaluation aims to demonstrate the outcome of technology in teaching and learning. These evaluation processes can be carried out simultaneously or at each stage of development or implementation (Alkhasawnh & Alqahtani, 2019; Guerra et al., 2016; Sasai, 2017). This study focused on formative evaluation to understand usability issues, challenges and factors influencing students' satisfaction with a mobile app.

Several related works have evaluated a self-regulated-based learning environment for supporting online learning experiences. Chang et al. (2022) examined the effects of online learning strategies on learning performance, self-regulation, and critical in a University online course. The study used a self-regulated flipped learning approach to experiment with whether the strategies could improve students' skills. The analysis used the analysis of covariance (ANCOVA). The result showed that students' skills were improved and recommended that further study can use massive data and mixed-method design to determine how a student's learning behaviour is affected. Similarly, Peng (2021) investigated college students' online self-regulation with the blended learning process using Effectiveness of Learning English (EOLE) and Online Self-Regulated English Learning (OSEL) survey instruments. The findings revealed that learners' self-evaluation, environment structuring, and goal setting could be used to interpret their English language learning effectiveness. These findings further highlight the importance of learners' self-evaluation, environment structuring, and goal setting in English language learning effectiveness.

Moreover, Manganello et al. (2019) evaluated the effectiveness of the self-regulated web-based learning platform among 418 students using the mixed methods. The result of the study provided evidence of engagement and active learning among students and that web-based self-regulated learning platforms can support students' active learning process and development of skills. While the evaluation results were useful, the study lacks a theoretical model to provide a lens through which results can be interpreted. Similarly, Alkhasawnh and Alqahtani (2019) explored the effects of using online pre-graduate courses focused on self-regulated learning strategies to improve student self-regulation and academic achievement among 70 students using two online courses to teach the same concepts. One group of students was taught online courses sponsored by self-regulated learning strategies, and the other group was taught online courses not funded by self-regulated learning strategies. The result demonstrated that the online course supported by self-regulated learning interventions substantially influences student self-regulation and academic outcomes.

Chumbley et al. (2018) investigated self-regulated learning skills among 100 students. The research used the online self-regulated learning questionnaire (OSLQ). The findings show that students have the highest degree of self-regulation within the dimension of environmental structuring and goal setting. The lowest level of self-regulation of learning was in the task strategies. Female students had a higher level of self-regulated learning while there was little difference in ethnicity. Low correlations were observed between students' interaction with online courses and perceived online self-regulated levels of their learning process. The research called for further work on hybrid dual enrollment courses to explore students' self-monitor learning process.

Moreover, Nussbaumer et al. (2015) evaluated how a web‐based service supports self‐regulated learning in virtual environments on 22 respondents. The results showed that the system supported the self-regulated learning process, promising user acceptance. Similarly, Furthermore, Kinnebrew et al. (2015) used a mixed-method study on 25 respondents to examine how the students use the learning environment to support the self-regulation process in SimSelf, an open-ended environment for science learning. The result showed that students who used the supporting tools had demonstrated adequate knowledge of the science topic. Conversely, students who did not use the tools effectively generally achieved minimal success at their learning tasks.

While several works have been conducted to evaluate a self-regulate-based learning environment, limited evaluation studies are based on a theoretical model. Furthermore, most evaluation studies are quantitative and lack a theory to interpret the findings. There is a scarcity of studies that integrated the information system success model constructs to understand factors responsible for user satisfaction moderated by the social cognitive model. A self-regulated smart learning environment is a new learning paradigm that needs to be examined from user experiences to understand its implementation issues to inform pedagogical design principles for future implementations.

## Research model

This study adopted the integration of the information system success model and social cognitive model to explore factors influencing students' satisfaction in a self-regulated smart learning environment (Gambo & Shakir, 2019). Previous studies have tested and validated these models (Awang et al., 2019; Yakubu & Dasuki, 2018; Mohammadi, 2015; Zalazar-Jaime & Medrano, 2021; Zakiah & Fajriadi, 2020). However, there is a scarcity of studies on integrating the two models and whether the constructs of a social cognitive model can moderate information system quality to influence user satisfaction. These models are briefly discussed as follows:

### Information system success model

The information system success model (DeLone & McLean, 2003) determines whether the system was successful, looking at the factors of information quality (information display, accuracy, relevancy, and completeness), system quality (system performance and response time, user interface quality (ease of use and user interface), reliability, security, and availability as well as service quality (quick response time and help desk), all of which were considered individually to be net benefits. These constructs can influence intention, user satisfaction, and the net benefit of using an information system (Gambo & Shakir, 2019).

### Socio-cognitive model

The social cognitive theory offers a learning model that considers the learner's social context and personal aspects like impact and cognition (Bandura, 2002; Bembenutty et al., 2016). Their cognitive processes and social context impact students' motivation. The learner's learning behaviour, such as using learning strategies and their environment, may influence their self-efficacy views. Peers (another environmental component) can impact students' self-efficacy and learning habits, leading to changes in the students' learning environment. Individuals' interpretations of their performance outcomes affect their circumstances and self-belief, affecting their subsequent performances (Bembenutty et al., 2016). Furthermore, the authors asserted that learners' were self-regulating when they were in charge of their own personal, behavioural, and environmental aspects.

This study adopted the constructs of the information system success model (system quality, information quality, and service quality & user satisfaction) and the constructs of the social cognitive model (behavioural, environmental & personal) to investigate factors influencing students' satisfaction in a self-regulated smart learning environment. This study hypothesized that the information system success model (system quality, information quality & service quality) directly influences user satisfaction, and the constructs of the social cognitive model (behavioural, environmental & personal) moderate factors influencing users' satisfaction.

**Fig. 9:**
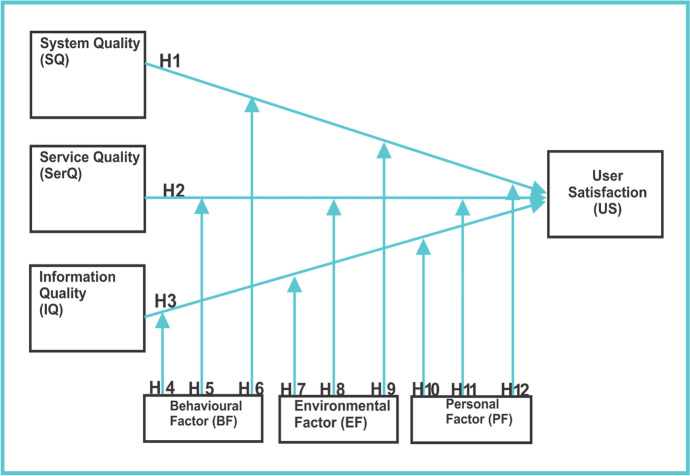
Model for evaluating self-regulated smart learning environment

Figure 9 shows the research model, and the categorization of the constructs is shown in Table 1.

**Table 1:** The categorization of the dimensions and associated constructs

Dimension	Constructs
Personal	Self‑efficacy, motivation, instincts, drives, traits, wisdom, thoughts, feelings, beliefs, self-perception, goals, intentions, and other motivational forces within the individual (Berkeley et al., 2019; Mohamad & Osman, 2018)
Behavioural	Time management, learning strategies, goal setting, help-seeking, and reflection (Berkeley et al., 2019; Cox et al., 2017; Yakubu & Dasuki, 2018)
Environmental	Family members, friends, tutors, and colleagues. size of a room, the ambient temperature, or the availability of resources (Berkeley et al., 2019; Mohamad & Osman, 2018)
Information Quality	Readable, accuracy, relevance & completeness (Awang et al., 2019; Yakubu & Dasuki, 2018)
System Quality	Response time, ease of use, user interface usability, responsiveness, adaptability & reliability (Awang et al., 2019; Devi et al., 2017)
Service Quality	Knowledge, empathy, responsiveness, and effectiveness of the software and support from the facilitator and peers (Devi et al., 2017; Yakubu & Dasuki, 2018)
User Satisfaction	Information system service, quality & system in terms of usefulness, support, and effectiveness (Berkeley et al., 2019; Yakubu & Dasuki, 2018)

Thus, based on these assumptions and propositions, the following hypotheses were stated:

### System Quality (SQ)

This is the extent to which the system can deliver benefits to the user in terms of response time, ease of use, user interface usability, responsiveness, adaptability & reliability (Awang et al., 2019; Devi et al., 2017; Lwoga, 2012). Findings from previous studies have shown that system quality has a positive relationship with user satisfaction (Berkeley et al., 2019; Cox et al., 2017; Mohamad & Osman, 2018; Mohammadi, 2015; Sasai, 2017). Consequently, this study hypothesizes that:

- **H1**: System quality will have a positive relationship with user satisfaction

### Service Quality (SerQ)

This is the extent of delivering benefits to the user regarding processing time, availability & support. In this study, the characteristics include responsiveness and effectiveness of the mobile app and support from the facilitator and peers. Findings from the previous have shown that service quality has a positive relationship with user satisfaction (Awang et al., 2019; Bembenutty et al., 2016; Mohammadi, 2015). Thus:

- **H2**: Service quality will have a positive relationship with user satisfaction

### Information Quality (I.Q.)

This is the quality of the content of *information* systems in terms of readability, accuracy, relevance & completeness (Yakubu & Dasuki, 2018). In this study, information quality is referred to the quality of the learning resources uploaded to the mobile app and evaluated by the students. These qualities include availability, ease of comprehension, relevance, completeness, and security. Findings from the previous studies have shown that information quality has a positive relationship with user satisfaction (Berkeley et al., 2019; Cox et al., 2017; Mohamad & Osman, 2018; Sasai, 2017). It is thus, hypothesized as:

- **H3**: Information quality will have a positive relationship with user satisfaction

### Behavioural Factor (BF)

This measures knowledge and skill to perform a given behaviour in a learning environment (Awang et al., 2019). The behavioural factors include time management, learning strategies, goal setting, help-seeking, and reflection (Berkeley et al., 2019; Cox et al., 2017; Mohamad & Osman, 2018; Sasai, 2017). Findings from the Previous studies have shown that users' knowledge and skills can facilitate satisfaction (Berkeley et al., 2019; Mohamad & Osman, 2018; Sasai, 2017). Thus, this study hypothesizes that:

- **H4**: Behavioral factor moderates the relationship between system quality and user satisfaction
- **H5**: Behavioral factor moderates the relationship between service quality and user satisfaction
- **H6**: Behavioral factor moderates the relationship between information quality and user satisfaction

### Environmental Factor (EF)

This refers to the factors affecting a person's social or physical environmental behaviours. Social environments include family members, friends, tutors, and colleagues. The physical environment is the size of a room, the ambient temperature, or the availability of resources (Berkeley et al., 2019). In this study, the environmental factors are peers and facilitators that can motivate students to learn using the mobile app. Findings from the previous studies have shown that environmental factors positively influence user intention and satisfaction in using learning technologies (Berkeley et al., 2019; Cox et al., 2017; Mohamad & Osman, 2018; Sasai, 2017). Thus, this study hypothesizes that:

- **H7**: Environmental factor moderates the relationship between system quality and user satisfaction
- **H8**: Environmental factor moderates the relationship between service quality and user satisfaction
- **H9**: Environmental factor moderates the relationship between information quality and user satisfaction

### Personal Factor (PF)

This is a personal characteristic in the form of instincts, self-efficacy, motivation, drives, traits, wisdom, thoughts, feelings, beliefs, self-perception, goals, intentions, and other motivational forces within the individual (Yakubu & Dasuki, 2018). This study includes experiences in online self-regulation, motivations, reflective mind, goal setting & strategic learning abilities. Personal characteristics can moderate learning satisfaction and shape the surrounding environment (Berkeley et al., 2019; Cox et al., 2017). Thus, this study hypothesizes that:

- **H10**: Personal factor moderates the relationship between system quality and user satisfaction
- **H11**: Personal factor moderates the relationship between service quality and user satisfaction
- **H12**: Personal factor moderates the relationship between information quality and user satisfaction

### User Satisfaction (US)

This is measured by how a user is contented with the information service, quality, and system; and is useful to support job delivery (Berkeley et al., 2019; Yakubu & Dasuki, 2018). The perception of individuals is relative to the expectation of using the system and has been incorporated in earlier studies (Mohammadi, 2015; Yakubu & Dasuki, 2018) and found to be influenced by the system quality antecedents.

## Methodology

### Study design

This study used cyclical mixed-method evaluations to understand students' experiences with the mobile app. The qualitative method explore factors influencing students' satisfaction with the mobile app. The qualitative used focus group discussion to explore usability issues and challenges in the mobile app implementations.

The first mixed-method evaluation was conducted in February 2020, and the Second evaluation took place in May 2020. The combined findings of the mixed-method form the first evaluation cycle, and these findings were used to improve the mobile app for the second mixed-method evaluation. This process can be extended into several evaluation cycles based on the iterative needs as required in the design-based research (Sandoval & Bell, 2004).

### Research instruments

An online survey instrument was employed in the quantitative approach. It was chosen as the most effective way of data collection to eliminate errors caused by data handling, enhance response rate, and save expenditures. The survey instrument was divided into two sections. Section A: demographic information consists of sex, age, and level of self-regulated learning experiences, and section B is a 26-items scale that uses a 5-point Likert-type response format that ranges from strongly (5) to strongly disagree (1) to evaluate factors influencing students' satisfaction in self-regulated smart learning environment adopted form (Cox et al., 2017; Devi et al., 2017; Berkeley et al., 2019; Mohamad & Osman, 2018; Mohammadi, 2015; Sasai, 2017; Yakubu & Dasuki, 2018). At the same time, the qualitative approach used a focus group discussion seeking to know students' understanding of the learning process, usability issues that needed improvement, and challenges in using the mobile app that addressed the research questions ii, iii, and iv.

The supervisory team reviewed the research instruments, and a pilot test was conducted among ten simple random students to ensure no errors on the instruments. Minor corrections were made to the survey and open-ended focused group questions based on the students' feedback. The research instruments were further submitted to the faculty research and ethical review committee for approval before conducting the actual research study.

### Sample and sampling process

There are exactly 100 students who volunteered to use the mobile app and participate in the study. All the volunteered students received an email linked to the survey. The email also explained the study's goals and stated that participation was voluntary. The responses were anonymous to preserve students' privacy. Also, students might quit the survey or not answer questions they didn't like.

The research employed the Modified Cochran Formula for a small sample size (Cochran, 1963) at 95 percent confidence with a half response rate to estimate the sizable sample size for the online survey. A sample of 85 responses was enough to validate the sample. For qualitative methods, six of the volunteered students were invited for the focus group discussion to understand if they understood the learning process in the mobile app, interface usability issues that improve to support online learning experiences, and challenges in using the mobile app. The qualitative sample size is enough to validate the population, and this is in full compliance with the literature that the average sample size for qualitative research can vary from 5 to 50 for a large population and 2 to 30 for a small population (Fugard & Potts, 2015; Guest et al., 2017).

### Data analysis

The online survey data were managed and analyzed using SPSS version 25. The data acquired from the survey were analyzed using the structural equation modelling (SEM) technique in two steps: The use of confirmatory factor analysis (CFA) to evaluate the reliability and validity of the measurement model and the application of the structural model to test hypotheses (Anderson & Gerbing, 1988).

The open-ended questions data (qualitative data) were analyzed using a thematic analysis approach to understand the respondents' voices, which includes analysis: familiarization with datasets, generating initial codes, searching for themes, reviewing themes, and renaming themes (Braun & Clarke, 2006) using QSR NVivo 12. The thematic analysis results were presented to the respondents to mitigate multiple realities and reduce research bias (Kaplan & Duchon, 1988). This process will give a better interpretation of the reality and the voice of the respondents. Multiple realities were minimized by discussing the analysis results with the research respondents (Kaplan & Duchon, 1988). The combined findings of the studies provided insight into an improved mobile app for supporting students' online learning experiences and informing decisions towards future implementations.

## Findings of evaluation I

### Respondents characteristics

Table 2 shows that 76.47% were male and 23.53% were female, which shows that most respondents are male. Furthermore, 18.82% of the respondents were within 18–25 years of age, 42.35% were within 26–30 years of age, 11.77% were within 31–35 years of age, 17.65% were within 36–40 years of age, and only 9.41% are 41 and above the age. This result means that most respondents were young adults and could use emerging learning technologies to support an online learning process. On the self-regulated learning experiences, 0.00% were very low, 1.18% were low, 35.29% were medium, 43.52% were high, and 20% were very higher experiences. This result means that most respondents have high self-regulated learning experiences to support their online learning experiences.

**Table 2:** Respondents characteristics

Demographic	Frequency	Percentage (%)
Gender		
Male	65	76.47
Female	20	23.53
Age		
18–25	16	18.82
26–30	36	42.35
31–35	10	11.77
36–40	15	17.65
41 & above	8	9.41
Level of self-regulated learning experiences		
Very Low	0	0.00
Low	1	1.18
Medium	30	35.29
High	37	43.52
Very High	17	20.00

### Scale validation and measurement model

Table 3 shows the construct measurement used in the online survey and a summary of the descriptive analysis for each of the constructs used in the research model. The research indicates that all constructs have positive value, as their respective averages are somewhat over 4. Cronbach's alpha was calculated for the observed responses to determine the internal reliability of the constructs. Cronbach's alpha values were greater than 0.70, within the acceptable ranges (George & Mallery, 2003).

**Table 3:** Descriptive analysis of the constructs

Construct	Mean	Standard deviation	Cronbach's alpha
Behavior Factor (BF):	4.082	0.802	0.868
Personal Factor (PF):	4.106	0.730	0.748
Environment Factor (EF)	4.041	0.810	0.851
System Quality (SQ)	4.054	0.855	0.854
Information Quality (IQ)	4.103	0.796	0.798
Service Quality (ServQ)	4.016	0.826	0.772
User Satisfaction (US)	4.067	0.857	0.828

The structural equation modelling was applied using a two-stage process to be consistent with the previous studies. These include the measurement of the model constructs and relationships using the maximum likelihood and goodness-of-fit to estimate and test the model's parameters (Yakubu & Dasuki, 2018). ServQ3 and IQ2 were removed from the service quality and information quality constructs to confirm a good fit between the model and the corresponding data.

Table 4 shows the recommended, and actual measurements for different values suggested in the literature (Hair et al., 2010; Kline, 2015). All indices are within the recommended values, as shown in Table 4, indicating a good model fit.

**Table 4:** Model fit indices

Fit index	Recommended value	Measurement model	Structural model
CFI	> 0.90	0.976	0.976
GFI	> 0.90	0.913	0.914
AGFI	> 0.80	0.804	0.804
RMSEA	< 0.08	0.047	0.047
RMSR	< 0.10	0.035	0.035
NFI	> 0.90	0.942	0.942

*CFI,* Comparative fit index; *GFI,* Goodness-of-fit index; *AGFI,* Adjusted goodness-of-fit; *RMSEA,* Root mean square error of approximation; *RMSR,* Root say squared residuals; *NFI,* Normed fit index

The convergent validity and discriminant validity tests were implemented to validate the scales. To evaluate convergent validity, Hair et al. (2010) recommended a CR value greater than 0.80; all average variance extracted (AVE) values greater than 0.50, and the AVE values less than their corresponding CR values. Table 5 indicates that convergent validity has been established because all three requirements have been met.

**Table 5:** Composite reliability, convergent validity, discriminant validity, and factor correlation matrix

	CR	AVE	MSV	ASV	ServQ	PF	IQ	BF	EF	SQ	US
ServQ	0.802	0.616	0.510	0.434	0.785						
PF	0.868	0.667	0.597	0.478	0.801	0.817					
IQ	0.894	0.637	0.587	0.407	0.733	0.701	0.798				
BF	0.829	0.656	0.551	0.444	0.805	0.800	0.801	0.810			
EF	0.824	0.676	0.610	0.428	0.765	0.755	0.715	0.725	0.822		
SQ	0.821	0.701	0.618	0.408	0.786	0.757	0.786	0.616	0.749	0.837	
US	0.828	0.617	0.616	0.429	0.624	0.765	0.785	0.689	0.775	0.527	0.785

The discriminant validity is evaluated based on the three criteria recommended by Hair et al. (2010): The average share variance (ASV) and maximum share variance (MSV) be lower than the AVE value, and the square root of the AVE should be greater than their corresponding correlation values (Bagozzi & Yi, 1988). Table 5 reveals that all of the discriminant conditions have been met.

The next is to evaluate the relationships between the constructs and hypotheses tested in the research model. Because the fit indices were all within the recommended ranges and the validity conditions were also met, this indicates that the data is well-matched with the model.

### Hypothesis testing and structural model

**Fig. 10:**
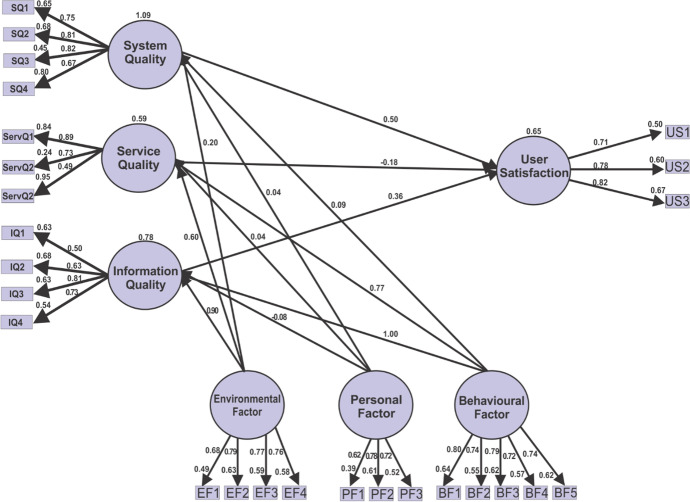
SEM analysis showing path coefficients, significance, and R.^2^

The confirmatory factor analysis evaluated the path's significance and strength to test the hypothesis and the structural model at a 0.05 level of significance. The strength of the relationship between the exogenous and endogenous variables (R^2^) was also measured. Figure 10 and Table 6 show the findings from the confirmatory factor analysis.

**Table 6:** Structural model and hypothesis testing

Hypothesis	Path			Estimate	P-value	Significance
H1	User Satisfaction	< –-	System Quality	0.50	***	S
H2	User Satisfaction	< –-	Service Quality	-0.18	***	S
H3	User Satisfaction	< –-	Information Quality	0.86	***	S
H4	System Quality	< –-	Behavioural	0.09	0.14	NS
H5	Service Quality	< –-	Behavioural	0.77	0.57	NS
H6	Information Quality	< –-	Behavioural	1.00	0.04	NS
H7	System Quality	< –-	Environmental	0.20	***	S
H8	Service Quality	< –-	Environmental	0.60	***	S
H9	Information Quality	< –-	Environmental	0.90	0.20	NS
H10	System Quality	< –-	Personal	0.40	0.20	S
H11	Service Quality	< –-	Personal	0.40	***	S
H12	Information Quality	< –-	Personal	-0.80	***	S

*NS,* Non-significant relationship; *S,* A significant relationship

Table 6 shows the path coefficients indicated all the twelve hypotheses tested; only eight were statistically significant (N): H1, H2, H3, H7, H8, H10, H11, and H12 supported students' satisfaction since their coefficients paths are less than their corresponding p-values. While H4, H5, 6, and H9 were not statistically significant (NS) because their coefficient paths were greater than their corresponding p-values. The finding revealed that the behavioural factors moderate all the information system qualities, i.e., service, system, and information. The result further revealed that personal factors moderate both system and service qualities, and only service quality impacted user satisfaction. These findings mean that a better service delivered by the information system can impact user satisfaction. Thus, the model's *R*^2^ values should be high for a minimum level of explanatory power (Götz et al., 2010). This study's *R*^2^ (0.65) values are shown in Fig. 10 and are high, which means that the model has a level of explanatory power.

The next subsections provide the findings of the focus group, which addressed implementation challenges, usability issues, and whether participants could follow the self-regulated learning process in the mobile app.

### Understanding of self-regulated learning process

The focus group discussions show that students understand the self-regulated learning process and support their online learning experiences. The ability of students to use the mobile app and whether it supports their online learning experiences identified the areas in which to be improved to enhance their learning experience. First, respondents stated that an attractive icon design plays an essential role in the self-regulated learning experience while installing the mobile app. One respondent said that even from the icon, icons usually attract people. So, the app's icon is essential for attraction to the learners. The interactive platform is also quite useful as students can put their queries on the forum and comment on questions. One respondent stated, "*I consider it helpful for a student considering the prototype's features. It allows the student to interact with another student, to comment*". The mobile also provides vast opportunities for the students to learn on various topics other than their mainstream courses. The discussion forum also motivates the students to learn. One respondent mentioned that "It supports and motivates *students to learn more with the help of the audio and video, presentations of slides, and a PDF file*". Moreover, learners can also monitor their performance regularly, so, in this sense, students can work to improve their weak areas with a variety of learning resources available. This result means students have a good grasp of the self-regulated learning process, and it could support their learning process to improve experiences.

### Usability of functionalities of the self-regulated smart learning environment that need improvement

Results show that some interface functionalities need to be improved to motivate and engage students in the learning process. Students identified four main areas related to improved functions of the mobile app. The first area is access to online libraries should be available in the functions of the smart learning environment. One of the students stated, "*You know we have those online libraries also. Maybe there should be a link for further research and stream of knowledge to support learning contents*". The second improvement is the background, and text colours should be improved. The participants noted the third usability issue is to provide a link to an online programming environment for practice.

Further, it was emphasized by the respondents that an interactive interface is most important where students could interact with their instructors; as mentioned by the respondent, "*there's something I would love to add. Suppose there would be an interface where the lecturer could interact with the students *via* the mobile app. In that case, I think maybe just a small interface that the students can communicate, ask questions and clarify something that they don't understand*". These observations are essential in the development process to improve students' experiences with an improved mobile app.

### Challenges in using self-regulated smart learning environment

The findings of the students' experiences indicated that there are inherent challenges associated with using the mobile app. While using the mobile app, students recognized various difficulties that need to be addressed. The issue of access to an affordable mobile device was emphasized. As students pointed out, while this mobile may be useful for the learning process, the costs and access to affordable mobile internet and smart devices could be a concern.

### Discussion

This study explored the experiences of students in the mobile app. The study used a mixed-method comprising a research model composed of six constructs (personal, behavioural, environmental, information quality, system quality & service Quality) and the open-ended focused group discussions to understand students' satisfaction. The result of the student's experiences in the mobile app provided insights into the implementation strategies to support online learning experiences.

Furthermore, the findings show that all the moderating factors (personal, behavioural & environmental) moderate information system constructs (system quality, service quality & information quality) except behavioural factors. This result means that students' facilitators and peers, the ambient temperature, time management, and availability of resources can support a student's online learning experiences and success (Berkeley et al., 2019; Navarro et al., 2014; Zalazar-Jaime & Medrano, 2021). These results were consistent with Alkhasawnh and Alqahtani (2019) findings, who found the impact of personal, behavioural & environmental factors but differ from the findings of Chumbley et al. (2018) on the behavioural factor. This result means that personal and environmental factors can moderate the extent of users' satisfaction with an information system.

Furthermore, all the information system constructs (service, system & information) directly influence students' satisfaction. These results were consistent with Berkeley et al. (2019); Yakubu and Dasuki (2018), who found information system constructs influence actual usage in learning management systems. The results support the need to develop and present an online learning environment that offers an active learning process with good reflective and visualization tools to support online learning experiences (Almuqrin & Mutambik, 2021; Navarro et al., 2014). The non-significant relationships between environmental and information quality, behavioural and system quality; behavioural and service quality, and behavioural and service quality indicated that information system constructs (service, quality & information) are not completely moderated by the behavioural and environmental factors, which is unexpected and contrary to the findings of the previous studies (Almuqrin & Mutambik, 2021; Berkeley et al., 2019; Zalazar-Jaime & Medrano, 2021). The students' learning strategies, peers and facilitators can moderate satisfaction (Almuqrin & Mutambik, 2021). The impact of the information system qualities on user satisfaction was 100% (1.06), 59% (0.59), and 78% (0.78) for system quality, information quality, and service quality, respectively. This result means that system quality has a high impact on user satisfaction compared to other qualities. Overall the impact of these information qualities and their moderating effects on user satisfaction is 65% (0.65), which is moderated high (Götz et al., 2010; Henseler et al., 2012; Urbach & Ahlemann, 2010).

The focus group discussion addressed the implementation challenges, usability issues, and understanding of the self-regulated smart learning process in the mobile app. The results revealed that students could follow the learning process in the mobile app and promote their online learning experiences. The students made several suggestions to improve their learning experiences, such as colour interface, more interactivity, the ability of the app to remember user login details, and interaction with peers and facilitators were suggested to improve the system. Furthermore, mobile network connectivity was identified as one of the challenges students face in using the mobile app. Among the factors influencing user satisfaction, personal factors seem to moderate all the quality antecedents and thus support students' motivation, engagement, beliefs, self-perception, and goal setting orientation to enhance learning experiences.

## Findings of evaluation II

### Respondents characteristics

Table 7 shows that 63.53% were male and 36.47% were female, which shows that most respondents were male. Furthermore, 16.57% of the respondents are within 18–25 years of age, 24.71% are within 26–30 years of age, 28.24% are within 31–35 years of age, 22.35% are within 36–40 years of age, and only 8.24% were 41 and above years of age. This result means that most of the respondents were young adults, and they can use emerging learning technologies to support an online learning process. On the self-regulated learning experiences, 2.35% were very low, 7.06% were low, 29.41% were medium, 47.06% were high, and 14.11% were very higher experiences. This result means that most respondents have high self-regulated learning experiences to support their online learning experiences.

**Table 7:** Respondents characteristics

Demographic	Frequency	Percentage (%)
Gender		
Male	54	63.53
Female	31	36.47
Age		
18–25	14	16.57
26–30	21	24.71
31–35	24	28.24
36–40	19	22.35
41 & above	7	8.24
Level of self-regulated learning experiences		
Very Low	2	2.35
Low	6	7.06
Medium	25	29.41
High	40	47.06
Very High	12	14.11

### Scale validation and measurement model

Table 8 displays the survey constructs and a summary of the descriptive analysis for each construct. All constructs get a favourable response, with averages somewhat above 4. The model variables' internal reliability was computed using Cronbach's alpha for the observed responses. All constructs had Cronbach's alpha values over 0.80, except BF, IQ, and the US, there were over 0.90 and rated excellent (George & Mallery, 2003).

**Table 8:** Descriptive analysis of the constructs

Construct	Mean	Standard deviation	Cronbach's alpha
Behavior Factor (BF):	4.212	0.932	0.929
Personal Factor (PF):	4.18	0.932	0.849
Environment Factor (EF)	4.23	0.947	0.894
System Quality (SQ)	4.25	0.955	0.888
Information Quality (IQ)	4.18	0.952	0.905
Service Quality (ServQ)	4.24	0.963	0.871
User Satisfaction (US)	4.27	0.982	0.927

The structural equation modelling was applied using a two-stage process to be consistent with similar studies (Yakubu & Dasuki, 2018). ServQ3 and IQ4 were removed from the service quality and information quality constructs to confirm a good fit between the model and the corresponding data.

Table 9 shows the recommended and actual measure model for various values (Hair et al., 2010; Kline, 2015). All indices were within the recommended values, as shown in Table 9, indicating a good model fit.

**Table 9:** Model fit indices

Fit index	Recommended value	Measurement model	Structural model
CFI	> 0.90	0.926	0.926
GFI	> 0.90	0.917	0.915
AGFI	> 0.80	0.842	0.848
RMSEA	< 0.08	0.077	0.076
RMSR	< 0.10	0.036	0.036
NFI	> 0.90	0.932	0.930

*CFI,* Comparative fit index; *GFI,* Goodness-of-fit index; *AGFI,* Adjusted goodness-of-fit; *RMSEA,* Root mean square error of approximation; *RMSR,* Root say squared residuals; *NFI,* Normed fit index

The convergent validity and discriminant validity tests were implemented to validate the scales. To evaluate convergent validity, Hair et al. (2010) recommended a CR value greater than 0.8; all average variance extracted (AVE) values greater than 0.50, and the AVE values less than their corresponding CR values. Table 10 shows that all three conditions have been met, thus establishing convergent validity.

**Table 10:** Composite reliability, convergent validity, discriminant validity, and factor correlation matrix

	CR	AVE	MSV	ASV	ServQ	PF	IQ	BF	EF	SQ	US
IQ	0.905	0.931	0.704	0.915	0.965						
BF	0.929	1.055	0.723	0.930	0.890	1.027					
PF	0.848	1.082	0.651	0.854	0.952	1.006	1.040	PF			
EF	0.892	1.055	0.673	0.895	0.906	1.027	0.991	1.027			
SQ	0.885	1.082	0.660	0.898	0.965	0.985	1.040	1.018	1.040		
ServQ	0.874	0.947	0.699	0.875	0.964	0.913	0.941	0.923	0.973	0.973	ServQ
US	0.930	0.933	0.816	0.935	0.897	0.886	0.966	0.867	0.917	0.818	0.966

To evaluate discriminant validity, Hair et al. (2010) suggested three conditions: The average share variance (ASV) and maximum share variance (MSV) should be less than their corresponding AVE values; the square root of the AVE should be greater than their corresponding correlation values (Bagozzi & Yi, 1988). Table 10 demonstrates that all discriminant conditions have been met based on the three conditions.

The next is to evaluate the relationships between model constructs and hypotheses testing. Because the fit indices are all within the recommended ranges and the validity conditions are also met, this indicates that the data is well-matched with the model.

### Hypothesis testing and structural model

**Fig. 11:**
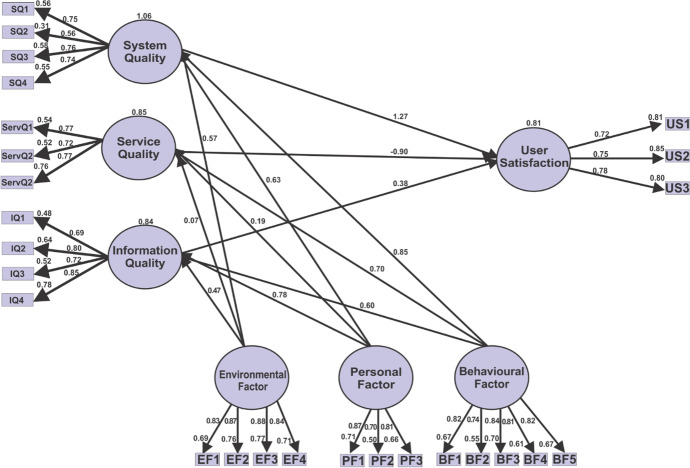
SEM analysis showing path coefficients, significance, and R^2^

The confirmatory factor analysis evaluated the path's significance and strength to test the hypothesis and the structural model. The strength of the relationship between the exogenous and endogenous variables (R^2^) was also measured. Figure 11 and Table 11 show the structural model and findings from the confirmatory factor analysis. The confirmatory factor analysis evaluated the path's significance and strength to test the hypotheses and the structural model at a 0.05 level of significance.

**Table 11:** Structural model and hypothesis testing

Hypothesis	Path			Estimate	P-value	Significance
H1	User Satisfaction	< –-	System Quality	1.7	0.01	NS
H2	User Satisfaction	< –-	Service Quality	-0.90	***	S
H3	User Satisfaction	< –-	Information Quality	0.38	0.16	NS
H4	System Quality	< –-	Behavior	0.85	***	S
H5	Service Quality	< –-	Behavior	0.70	0.37	NS
H6	Information Quality	< –-	Behavior	0.60	0.45	NS
H7	System Quality	< –-	Environment	0.57	***	S
H8	Service Quality	< –-	Environment	0.67	***	S
H9	Information Quality	< –-	Environment	0.47	***	S
H10	System Quality	< –-	Personal	0.63	***	S
H11	Service Quality	< –-	Personal	0.19	***	S
H12	Information Quality	< –-	Personal	0.78	***	S

*NS,* Non-significant relationship, *S,* A significant relationship

The path coefficients indicated all the twelve relationships tested; only seven were statistically significant (S): H2, H4, H7, H8, H9, H10, H11, and H12 supported students' satisfaction since their coefficients paths were less than their corresponding p-values. While H1, H3, H5, and H6 were not significant (N.S.) because their coefficients path were greater than their corresponding p-values. The finding revealed that all the information system qualities, i.e., service, system, and information influences, directly influence user satisfaction. This result means the qualities of the information system (mobile app) impact students' satisfaction.

On the other hand, information system qualities are moderated by behavioural, personal, and environmental. The impact of these moderators has indirect influences on students' satisfaction. The coefficient of determination *R*^2^ of each model construct was used to evaluate the structural model. Thus, the model's *R*^2^ values should be high for a minimum level of explanatory power (Götz et al., 2010). The *R*^2^ (0.81) values of this study are shown in Fig. 11 are high, which means that the model has a level of explanatory power.

The next subsections provide the findings of the focus group, which addressed implementation challenges, usability issues, and whether participants could follow the self-regulated learning process in the mobile app.

### Understanding of self-regulated learning process

The focus group discussions show that students understand the self-regulated learning process and support their online learning experiences. Students identified the areas in which the mobile app needs to be improved to enhance their learning experience. First, respondents stated that the discussion forum is easy to access and use while using it for their learning experiences. The self-regulated smart learning environment provides a conducive learning environment. One respondent stated that "*it supports learning because of a motivated and conducive environment. Moreover, visualizations also play a very important role and facilitate the students' learning experiences*". A self-regulated learning environment also provides wide opportunities for the students to learn on various topics other than their mainstream courses. These platforms also motivate the students to learn, as one respondent mentioned that they "*support and motivate students to learn more with the help of the audio and video, presentations of slides, and a PDF file*". This result means respondents have a good grasp of the learning process in the self-regulated learning process and believe that it supports their learning process for improving learning experiences.

### Usability functionalities of the self-regulated smart learning environment that need improvement

The findings show that some interfaces need to be improved to motivate and engage students in the learning process. Students identified four issues related to improving the functionalities and interface of the mobile. First is an online library on related subjects to support the learning process. One of the respondents stated, "*You know we have online resources, like a library and other valuable resources; maybe there should be a link for further research*". Secondly, some text colour needs to be visible and attractive to enhance readability. Thirdly, more courses should support various skills in different domains. One of the respondents stated that "*if more courses can be added to support various learning skills will be fine*". Fourthly, the need for a demo at the beginning of each course to enable a new user to understand the self-regulated learning process will be helpful. These observations are essential in the development process to provide feedback from the students' experiences to improve the mobile app supporting online learning experiences.

### Challenges in using self-regulated smart learning environment

Results from students' experiences show inherent challenges based on the locations. Respondents identified multiple challenges while using the mobile app. No doubt, self-regulated learning platforms play an essential role in the learning process, but it also needs durable and high-speed access to the internet to access these resources. So, the poor network connection was the primary challenge, as mentioned by the respondents. The mobile app can be accessed from smartphones or laptops only, so affordability to these devices is also a challenge. Also, not all students can afford a smartphone in our environment here, so the organization or the government can provide a smart program for smart learning. Students faced a sudden interruption in the platform's function, so the mobile app's features should be improved to avoid creating problems for the learners while learning. The mobile app should also be available on other operating systems; as students are using different devices, they could also access the app in their operating system. One of the challenges facing the developing world is access to good networks, even in rural communities. The provision of subsidized smart mobile devices with the re-installed app can further support the students in the online learning process.

### Discussion

This study explored the experiences of students on mobile. The result shows that all the moderating factors (personal, behavioural & environmental) moderate information system constructs (system quality, service quality & information quality) except information quality. This result means that students' self-motivation, collaborative skills, time management, and availability of resources can support online learning experiences and success (Navarro et al., 2014; Zalazar-Jaime & Medrano, 2021). Furthermore, all the information system constructs (service, system & information) directly influence students' satisfaction. These results were consistent with Berkeley et al. (2019) and Yakubu and Dasuki (2018), who found information system constructs influence actual usage in learning management systems. The results support the need to develop and present an online learning environment that is motivated and engaging with a good user interface and information display to support online learning experiences (Almuqrin & Mutambik, 2021; Yakubu & Dasuki, 2018). The non-significant relationships between behavioural and information quality; behavioural and system; environment and information quality; environment and service quality mean that information system constructs (service, quality & information) indicated that student's satisfaction was not moderated by the behavioural and environment, which is contrary to the previous studies (Almuqrin & Mutambik, 2021; Zalazar-Jaime & Medrano, 2021). Students' time management, learning strategies, peers, teachers, and family can moderate satisfaction (Almuqrin & Mutambik, 2021; Berkeley et al., 2019). The impact of the information system qualities on user satisfaction is 100% (1.06), 85% (0.85), and 84% (0.84) for system quality, service quality, and information quality, respectively. This result means that system quality has a high impact on user satisfaction compared to other qualities. Overall the impact of these information qualities and their moderating effects on students' satisfaction is 81% (0.81), which is a high (Götz et al., 2010; Henseler et al., 2012; Urbach & Ahlemann, 2010). The student's high level of SRL experience had been increased by 3.6% from the first evaluation to the second evaluation. This result means that the mobile app has supported students' levels of SRL experiences.

The focus group discussion addressed the implementation challenges, usability issues, and understanding of the self-regulated smart learning process in the mobile app. The results revealed that students could follow the learning process in the mobile app and promote their online learning experiences. However, few suggestions have been suggested for incorporation, such as adding a platform for online coding, offline capability for limited network issues, and a message to confirm the sent message during interactions. Furthermore, mobile network connectivity was identified as one of the challenges facing students' satisfaction. These results supported the need to improve the quality of the learning resources uploaded and students' interactions among peers and facilitators to enhance online learning experiences.

## Conclusion

There is a lack of research on students' experiences in a self-regulated smart learning environment to understand the implementation of online learning experiences. This study used cyclical mixed-method evaluations to understand students' experiences with the mobile app. The findings of this evaluation will inform pedagogical design principles for implementing the self-regulated smart learning environment.

In the first evaluation, findings show that only eight were statistically significant. The moderating factors moderate all the constructs of the information system except behavioural factors. There was a statistically significant association between information system constructs and satisfaction. This result is expected as an online learning environment that is motivated and engaging with a good user interface, and information display can provide students' satisfaction. Overall, the impact of these information qualities and their moderating effects on user satisfaction was high, which is good explanatory power. Some issues, such as improvements in user interactions, online coding facilities, reference links, and low internet connectivity, are identified to improve the mobile app and challenges to support effective implementation.

The second evaluation shows that only seven of the constructs were statistically significant. The moderating factors moderate all the constructs of the information system except information quality. According to the findings, there was a statistically significant relationship between information system constructs and students' satisfaction. This finding is predicted, as the results demonstrate that presenting an online learning environment that is motivating and engaging and having a usable user interface and information display can increase students' satisfaction. Students' satisfaction was highly influenced by these information qualities and their moderating effects, indicating strong explanatory power. Some issues such as improvements in user interactions, offline functionality, web-based version, subsidized smart device, and low internet connectivity were identified to improve the mobile app to support online learning experiences.

The student's high level of SRL experience had been increased by 3.60% from the first evaluation to the second evaluation. Furthermore, the student's level of satisfaction had increased by 0.16% from the first evaluation to the second evaluation. This means that the implementation of the mobile app has impacted students' experiences**.**

In conclusion, higher education leaders and educators should take steps to guarantee that the self-regulated smart learning environment is dependable, simple to use, and available. It is also the administrators' responsibility to guarantee that teachers are instructed on how to utilize the system effectively to guarantee that the information/resources placed on the system are accurate. As a result, proper training on using the system should be provided. There should add support for the self-regulated smart learning environment, particularly for the novice student to support online learning experiences.

## Research and practices implications

The penetration of digital technologies in developed countries is sufficient to use applications technology in educational institutions and other related organizations. While developing countries are still lagging in bridging the digital divide, the relevance of this study mandates the building of a better wireless network and the deployment of a subsidized approach and engaging learning content to improve student's learning outcomes. The challenges posed by the Covid-19 pandemic call for a new policy for online education and instructional training and development. The new curriculum must be developed to meet the yearning and aspirations of a new breed of digital learners. Using a self-regulated smart learning environment is an investment in the development, deployment, and training. This transformation calls for actions from the educational stakeholders to address issues for the successful use of such applications in an educational institution.

## Limitations of the study and further works

Implementing the self-regulated smart learning environment has shown that it can support students' online learning experiences. However, there is a call for improving interactivity and the availability of mobile internet services. Furthermore, the study calls for other versions of the application or specifically a web-based version that can support both online and offline users.

The research sample was drawn from a single department at Adamawa State University in Mubi, Nigeria. Thus, the findings from this study can't be generalized to reflect the experience of a student in the self-regulated smart learning environment among the population of the University. Furthermore, the learning environment only used one course to implement the learning process. There is a need for future research to involve other departments, universities, and courses to understand the applicability of the application in supporting the online learning process. The study only integrates the information system model and social cognitive theory constructs and considers only constructs that influence user satisfaction. There is a need in the future to investigate other models and constructs to see if these can influence user satisfaction for developing a broader policy framework for the future development of such a system.

## Section A: Demographic information

(Please fill appropriate)

## Section B: Students' experiences in the smart learning environment

(Please check ONE that describes you)

**Table:**

**Behaviour Factor (BF)**: Knowledge and skill to perform a given behaviours	Strongly Disagree	Disagree	Neutral	Agree	Strongly Agree
[1] I set a goal of a self-regulated smart learning environment to help me manage the learning process					
[2] I use specific learning content in a self-regulated smart learning environment to reinforce my learning process					
[3] I seek help from peers or facilitators to help me make progress					
[4] I set time to achieve my learning goal in a self-regulated smart learning environment					
[5] I monitor and reflect on my learning progress in the self-regulated smart learning environment					
**Personal Factor (PF)**: The behavioural deposition of the form of instincts, self-efficiency, motivation)	Strongly Disagree	Disagree	Neutral	Agree	Strongly Agree
[6] I have self-efficiency to achieve my personal learning goal in the self-regulated smart learning environment					
[7] I have a self-believe in achieving learning progress in the self-regulated smart learning environment					
[8] I am motivated to use a self-regulated smart learning environment					
**Environment Factor (EF): R**efers to the factors that can affect a person's behaviours (social & Physical)	Strongly Disagree	Disagree	Neutral	Agree	Strongly Agree
[9] I receive support from peers and facilitators in learning in the self-regulated smart learning environment					
[10] Although we don't have to attend daily classes, I still try to distribute my studying time evenly across days					
[11] The feedback I received on learning progress in the self-regulated smart learning environment is motivating					
[12] I used my smart mobile devices for learning at any time					
**System Quality (SQ):** Is the extent to which the system can deliver benefits to the user (response time, ease of use, user interface, & reliability)	Strongly Disagree	Disagree	Neutral	Agree	Strongly Agree
[13] the self-regulated smart learning environment is available most of the time					
[14] the self-regulated smart learning environment is highly reliable with minimal downtime					
[15] The response time of a self-regulated smart learning environment is easy to use					
[16] The user interface in a self-regulated smart learning environment is interactive and friendly					
**Information Quality (IQ):** is the *quality* of the content of *information* systems (readable, accuracy, relevance & completeness)	Strongly Disagree	Disagree	Neutral	Agree	Strongly Agree
[17] Overall, information displayed from a self-regulated smart learning environment is complete					
[18] Overall, the information displayed from a self-regulated smart learning environment is readable					
[19] Overall, the information I get from the self-regulated smart learning environment is accurate					
[20] Overall, the information from the self-regulated smart learning environment is relevant					
**Service Quality (SQ):** Assessment of how well a delivered *service* conforms to the user's expectations (processing time, availability, & support)	Strongly Disagree	Disagree	Neutral	Agree	Strongly Agree
[21] The self-regulated smart learning environment is available anytime for learning					
[22] The self-regulated smart learning environment has a fast processing time					
[23] The support in a self-regulated smart learning environment helpful for learning					
**User Satisfaction (US):** The extent to which a user is pleased or contented with the information system (Service, quality & system	Strongly Disagree	Disagree	Neutral	Agree	Strongly Agree
[24] Overall, the service in a self-regulated smart learning environment is satisfied					
[25] Overall, the information displayed in the self-regulated smart learning environment is satisfied					
[26] Overall, the quality of a self-regulated smart learning environment is satisfied					

## Declarations

All data generated or analysed during this study can be requested.
